# Coordinated Plasticity among Glutamatergic and GABAergic Neurons and Synapses in the Barrel Cortex Is Correlated to Learning Efficiency

**DOI:** 10.3389/fncel.2017.00221

**Published:** 2017-07-26

**Authors:** Xin Zhao, Li Huang, Rui Guo, Yulong Liu, Shidi Zhao, Sudong Guan, Rongjing Ge, Shan Cui, Shirlene Wang, Jin-Hui Wang

**Affiliations:** ^1^Department of Pathophysiology, Bengbu Medical College Bengbu, China; ^2^Laboratory of Brain and Cognitive Science, Institute of Biophysics and University of Chinese Academy of Sciences Beijing, China; ^3^University of Chinese Academy of Sciences Beijing, China; ^4^Department of Psychiatry and Behavioral Sciences, Northwestern University, Feinberg School of Medicine Chicago, IL, United States; ^5^School of Pharmacy, Qingdao University Qingdao, China

**Keywords:** learning, memory, glutamate, GABA, neuron, synapse and barrel cortex

## Abstract

Functional plasticity at cortical synapses and neurons is presumably associated with learning and memory. Additionally, coordinated refinement between glutamatergic and GABAergic neurons occurs in associative memory. If these assumptions are present, neuronal plasticity strength and learning efficiency should be correlated. We have examined whether neuronal plasticity strength and learning efficiency are quantitatively correlated in a mouse model of associative learning. Paired whisker and odor stimulations in mice induce odorant-induced whisker motions. The fully establishment of this associative memory appears fast and slow, which are termed as high learning efficiency and low learning efficiency, respectively. In the study of cellular mechanisms underlying this differential learning efficiency, we have compared the strength of neuronal plasticity in the barrel cortices that store associative signals from the mice with high vs. low learning efficiencies. Our results indicate that the levels of learning efficiency are linearly correlated with the upregulated strengths of excitatory synaptic transmission on glutamatergic neurons and their excitability, as well as the downregulated strengths of GABAergic neurons' excitability, their excitatory synaptic inputs and inhibitory synaptic outputs in layers II~III of barrel cortices. The correlations between learning efficiency in associative memory formation and coordinated plasticity at cortical glutamatergic and GABAergic neurons support the notion that the plasticity of associative memory cells is a basis for memory strength.

## Introduction

Associative learning is a common approach for information acquisition and associative memory is essential to cognition (Wasserman and Miller, 1997; Suzuki, 2008; Wang and Cui, 2017). In terms of cellular mechanisms underlying associative learning and memory, associative memory cells are recruited in sensory cortices (Wang et al., 2014, 2015; Gao et al., 2016; Vincis and Fontanini, 2016), and their downstream brain regions (Takehara-Nishiuchi and McNaughton, 2008; Viskontas, 2008; Cai et al., 2016). In the meantime, neuronal plasticity occurs during associative memory (Honey and Good, 2000; Blair et al., 2001; Christian and Thompson, 2003; Jones et al., 2003; Silva, 2003; Zhang et al., 2004; Dityatev and Bolshakov, 2005; Fanselow and Poulos, 2005; Weeks et al., 2007; Frey and Frey, 2008; Neves et al., 2008; Nikitin et al., 2008; Wesson et al., 2008; Harlow et al., 2010; Rosselet et al., 2011; Cheetham et al., 2012; Margolis et al., 2012; Yan et al., 2016). The associative memory cells encode the newly acquired signal alongside innate signal for their integrations and storages, while the plasticity of associative memory cells may be involved in the efficiency of associated information acquisition and the strength of information retrieval and memory presentation (Wang et al., 2015; Gao et al., 2016; Yan et al., 2016; Guo et al., 2017; Wang and Cui, 2017). If neural plasticity is involved in the efficiency of information acquisition and memory presentation, the strength of neural plasticity should be correlated with the efficiency of associative learning and memory, which we aim to examine through this manuscript.

An interactive balance between excitatory and inhibitory neurons are essential for programming the brain codes to manage the well-organized cognitions (Ascoli et al., 2008). How the recruited associative memory cells including glutamatergic and GABAergic neurons (Yan et al., 2016) are refined coordinately for the storage and retrieval of the associated signals remains to be addressed in mammalians (Bienvenu et al., 2012; Letzkus et al., 2012). A current report demonstrates that excitatory synaptic transmission on glutamatergic neurons and their excitability are upregulated as well as that the excitability of GABAergic neurons and their input and output synaptic transmission are downregulated in the somatosensory cortices during associative memory (Yan et al., 2016). Whether their coordinated plasticity is correlated with the efficiency of associative learning and memory needs to be elucidated.

To better understand these questions, we have analyzed the correlation between learning efficiency and plasticity strength at barrel cortical glutamatergic and GABAergic neurons in a mouse model of associative memory, i.e., odorant-induced whisker motion (Wang et al., 2015; Gao et al., 2016; Yan et al., 2016; Guo et al., 2017). In order to read out cell-specific mechanisms, glutamatergic neurons were genetically labeled by yellow fluorescent protein, and GABAergic neurons were labeled by green fluorescent protein in the mice (Zhang et al., 2013). Cellular electrophysiology in these neurons in barrel cortical layers II~III in coronal section of brain slices were conducted to analyze refinements at these synapses and neurons.

## Materials and methods

All experiments were performed in accordance with the guidelines by the Administration Office of Laboratory Animals in Beijing, China. All experiment protocols were approved by Institutional Animal Care Unit Committee in the Administration Office of Laboratory Animals in Beijing, China (B10831).

### Mouse model of associative memory

To analyze cell-specific mechanism for associative memory we used C57 Thy1-YFP/GAD67-GFP mice (Zhang et al., 2013) whose glutamatergic neurons were genetically labeled by yellow fluorescent protein (YFP) and GABAergic neurons were labeled by green fluorescent protein (GFP).

Mice with similar odorant sensitivity and whisker motion in postnatal day 20 were classified into two groups and trained by the simultaneous pairing of mechanical whisker stimulus (WS) with odor stimulus (OS, butyl acetate toward the noses), or the unpairing of these stimuli (control) (Wang et al., 2015; Gao et al., 2016; Yan et al., 2016). The paired and unpaired WS and OS were given by multiple-sensory modal stimulator (MSMS, pattern number 201410499466), in which the intensities, time and intervals of OS and WS were precisely set. WS was given by a fine mechanical bar (1 mm in the diameter) to make longer whiskers being fluctuated passively, in which the intensity and frequency were precisely controlled by the MSMS. OS was given by a liquid drop of butyl acetate at the tip of fine tube (0.5 mm in the diameter) toward the front of the noses, which was also controlled by this MSMS (please refer video one in our previous publication; Wang et al., 2015). OS intensity was sufficient to induce the response of olfactory bulb neurons as seen by two-photon Ca^2+^ imaging (Wang et al., 2015). WS intensity was sufficient to evoke whisker fluctuation after WS ended. Each of the mice was trained 20 s in each time, five times per day with 2 h of intervals for 10 days. During the training, each mouse was placed in a home-made cage, in which the body and limbs were allowed to move freely though the running was restricted. Cares were taken to include no stressful experiment conditions nor circadian disturbance to the mice that showed normal whisking and symmetric whiskers (Wang et al., 2015; Gao et al., 2016; Yan et al., 2016). Long whiskers (such as arcs 1~2) on the same side and rows were assigned for mechanical stimulation and observation in the odor-test. This selection was based the studies of cross-modal plasticity (Ni et al., 2010; Ye et al., 2012). We did not trim short whiskers since whisker trimming elevated the excitability of the barrel cortex (Zhang et al., 2013).

Whisker motion tracks were monitored by a digital video camera (240 Hz) and were quantified in whisking angles and frequency (MB-Ruler, version 5.0 by Markus Bader, MB-Softwaresolution, Germany). The responses of mouse whiskers to the odor-test (butyl acetate, 20 s) were measured before the training and at the end of each training day to quantify the onset time and levels of conditioned reflex (CR). Odorant-induced whisker motion was also quantified based on whisking frequency and whisking angles. Whisking frequency was the fluctuation times of long whiskers per second (Hz), and whisking angles were the motion angles from the resting position to fluctuation-end position. CR-formation was defined to meet the following criteria. The patterns of odorant-induced whisker motion were similar to those of WS-induced whisker motion, but not spontaneous whisking. The whisking frequency and whisking angles significantly increased, compared to control and before the training. As this type of whisker motion induced by odorant was originally induced by WS, the odor signal initiated a recall of whisker signal and then led to whisker motion (Wang et al., 2015; Gao et al., 2016; Yan et al., 2016).

Learning efficiency was merited based on the time at the fully establishment of odorant-induced whisker motion. According to our data from all of the mice in the expression of odorant-induced whisker motion, we defined learning ability as either high or low efficiency. If odorant-induced whisker motion reached to a plateau level before or at training day 6, the mice were characterized as high learning efficiency (HLE). If odorant-induced whisker motion reached to the plateau level at training day 10 or after, the mice were characterized as low learning efficiency (LLE). For the mice with high learning efficiency, there was no statistical difference for the level of odorant-induced whisker motion in training day 6 vs. training day 10, though their values were different. In other words, data points with no further statistical change in the level of odorant-induced whisker motion were thought to be the plateau level. In this study, when we plotted learning curve for individual mice, we found their learning curves tending to be these two groups. No mice were excluded in data analysis.

### Brain slices and neurons

Cortical slices (400 μm) were prepared from the mice of CR-formation and unpaired controls. They were anesthetized by inhaling isoflurane and decapitated by a guillotine. The slices were cut by Vibratome in the oxygenated (95%O_2_/5%CO_2_) artificial cerebrospinal fluid (ACSF), in which the chemical concentrations (mM) were 124 NaCl, 3 KCl, 1.2 NaH_2_PO_4_, 26 NaHCO_3_, 0.5 CaCl_2_, 4 MgSO_4_, 10 dextrose, and 5 HEPES, pH 7.35 at 4°C. The slices were held in the oxygenated ACSF (124 NaCl, 3 KCl, 1.2 NaH_2_PO_4_, 26 NaHCO_3_, 2.4 CaCl_2_, 1.3 MgSO_4_, 10 dextrose, and 5 HEPES, pH 7.35) at 25°C for 2 h. The slices were transferred to submersion chamber (Warner RC-26G) that was perfused with the oxygenated ACSF at 31°C for whole-cell recording (Wang and Kelly, 2001).

Electrophysiological recordings on the neurons in layers II–III of barrel cortices of brain slices, which were on the contralateral side of trained whiskers, were conducted under DIC-fluorescent microscope (Nikon FN-E600, Japan). These brain slices including barrel cortices were the coronal sections of mouse brains with the emergence of the dorsal hippocampus, which corresponded to the projection areas of longer whiskers. The wavelength at 488 nm excited GFP, and the wavelength at 575 nm excited YFP. GABAergic neurons showed basket shape and fast spiking with less adaptation in spike amplitudes and frequency (McKay and Turner, 2005; DeFelipe et al., 2013; Lu et al., 2014). Glutamatergic neurons showed pyramidal shape and regular spikes with the adaptation of spike amplitudes and frequency (Xu et al., 2015). Cerebral slices were coronal sections including the barrels correspondent to the projection from long whiskers that were stimulated in pairing WS and OS training.

### Whole-cell recording

Cortical neurons were recorded by MultiClamp-700B amplifier in voltage-clamp for their synaptic activities. Electrical signals were inputted into pClamp-10 (Axon Instrument Inc, CA USA) for data acquisition and analyses. Output bandwidth in this amplifier was 3 kHz. The pipette solution for studying excitatory synapses included (mM) 150 K-gluconate, 5 NaCl, 5 HEPES, 0.4 EGTA, 4 Mg-ATP, 0.5 Tris-GTP, and 5 phosphocreatine (pH 7.35; Ge et al., 2011, 2014). The solution for studying inhibitory synapses contained (mM) 130 K-gluconate, 20 KCl, 5 NaCl, 5 HEPES, 0.5 EGTA, 4 Mg-ATP, 0.5 Tris–GTP, and 5 phosphocreatine (Zhang et al., 2012). Pipette solutions were freshly made and filtered (0.1 μm), osmolarity was 295~305 mOsmol, and pipette resistance was 5~6 MΩ.

The functions of GABAergic neurons were assessed based on their active intrinsic properties and inhibitory outputs (Wang, 2003). The functional state of their inhibitory outputs was assessed by recording spontaneous inhibitory postsynaptic currents (sIPSC) under the voltage-clamp on glutamatergic neurons in the presence of 10 μM 6-Cyano-7-nitroquinoxaline-2,3-(1H,4H)-dione (CNQX) and 40 μM D-amino-5-phosphonovanolenic acid (D-AP5) in the ACSF to block ionotropic glutamate receptors (Wei et al., 2004; Ma et al., 2016a). Ten micromolars bicuculline was washed onto the slices at the end of experiments to test whether synaptic responses were mediated by GABA_A_R, which blocked sIPSCs in our experiments. The series and input resistances in all of the neurons were monitored by injecting hyperpolarization pulses (5 mV/50 ms), and calculated by voltage pulses vs. instantaneous and steady-state currents. It is noteworthy that pipette solution with the high concentration of chloride ions makes the reversal potential to be −42 mV. sIPSCs will be inward when the membrane holding potential at −65 (Wei et al., 2004; Xu et al., 2015; Wang G. Y., et al., 2016).

The functions of glutamatergic neurons were assessed based on their active intrinsic property and excitatory outputs (Wang, 2003). The functional state of their excitatory output was assessed by recording spontaneous excitatory postsynaptic currents (sEPSC) on GABAergic or glutamatergic neurons in presence of 10 μM bicuculline in ACSF to block ionotropic GABA receptors (Wang, 2003). Ten micromolars CNQX and 40 μM DAP-5 were added into the ACSF perfused onto the slices at the end of experiments to examine whether synaptic responses were mediated by GluR, which blocked EPSCs in our experiments. The series and input resistances for all cells were monitored by injecting hyperpolarization pulses (5 mV/50 ms), and calculated by voltage pulses vs. instantaneous and steady-state currents.

Action potentials at these cortical neurons were induced by injecting depolarization pulses, whose intensity and duration were altered based on the aim of the experiments. The ability to convert excitatory inputs into digital spikes was evaluated by input-outputs (spikes vs. normalized stimuli) when various stimuli were given (Chen et al., 2006a,b, 2008; Wang et al., 2008), in which stimulus intensities were step-increasing by 10% normalized stimulations. As the excitability of different neurons was variable such that step-increased depolarization pulses were given based on their normalization. The base value of stimulus intensity for this normalization at each neuron was the threshold intensity of depolarization pulse (1,000 ms in duration) to evoke a single spike (Chen et al., 2006b). We did not measure the rheobase to show the neuronal excitability, since this strength-duration relationship was used to indicate the ability to fire a single spike.

The recording of spontaneous synaptic currents, instead of the evoked synaptic currents, is based on the following reasons. sEPSC and sIPSC amplitudes represent the responsiveness and the densities of postsynaptic receptors. The frequencies imply the probability of transmitter release from an axon terminal and the number of presynaptic synapses innervated on the recorded neuron (Zucker and Regehr, 2002; Stevens, 2004). These parameters can be used to analyze presynaptic and postsynaptic mechanisms about neural interaction and plasticity. The evoked postsynaptic currents cannot separate these mechanisms. We did not add TTX into the ACSF to record miniature postsynaptic currents since we had to record neuronal excitability (Xu et al., 2015; Ma et al., 2016b).

Data were analyzed if the recorded neurons had the resting membrane potentials negatively more than −60 mV and action potential amplitudes more than 90 mV for GABAergic neurons as well as negatively more than −70 mV and action potential amplitudes more than 100 mV for glutamatergic neurons (Chen et al., 2008; Wang et al., 2008). The criteria for the acceptance of each experiment also included <5% changes in resting membrane potential, spike magnitude, and input resistance throughout each experiment. Input resistance was monitored by measuring cellular responses to hyperpolarization pulse at the same values as the depolarization that evoked action potentials. In order to estimate the effects of associative learning on neuronal spikes and synaptic transmission, we measured sEPSC, sIPSC, input-output under the conditions of control and associative memory, which were presented as mean ± SE.

### Statistical analyses

The paired *t*-test was used in the comparisons of the experimental data before and after associative learning, as well as the neuronal responses to whisker stimulus and odorant stimulus in each of the mice. One-way ANOVA or two-way ANOVAs were applied to make statistical comparisons in the changes of neuronal and synaptic activities among control mice, CR-formation mice with high learning efficiency and CR-formation mice with low learning efficiency.

## Results

### Pairing whisker and odor signals leads to odorant-induced whisker motion with different efficiency

**Figure 1:**
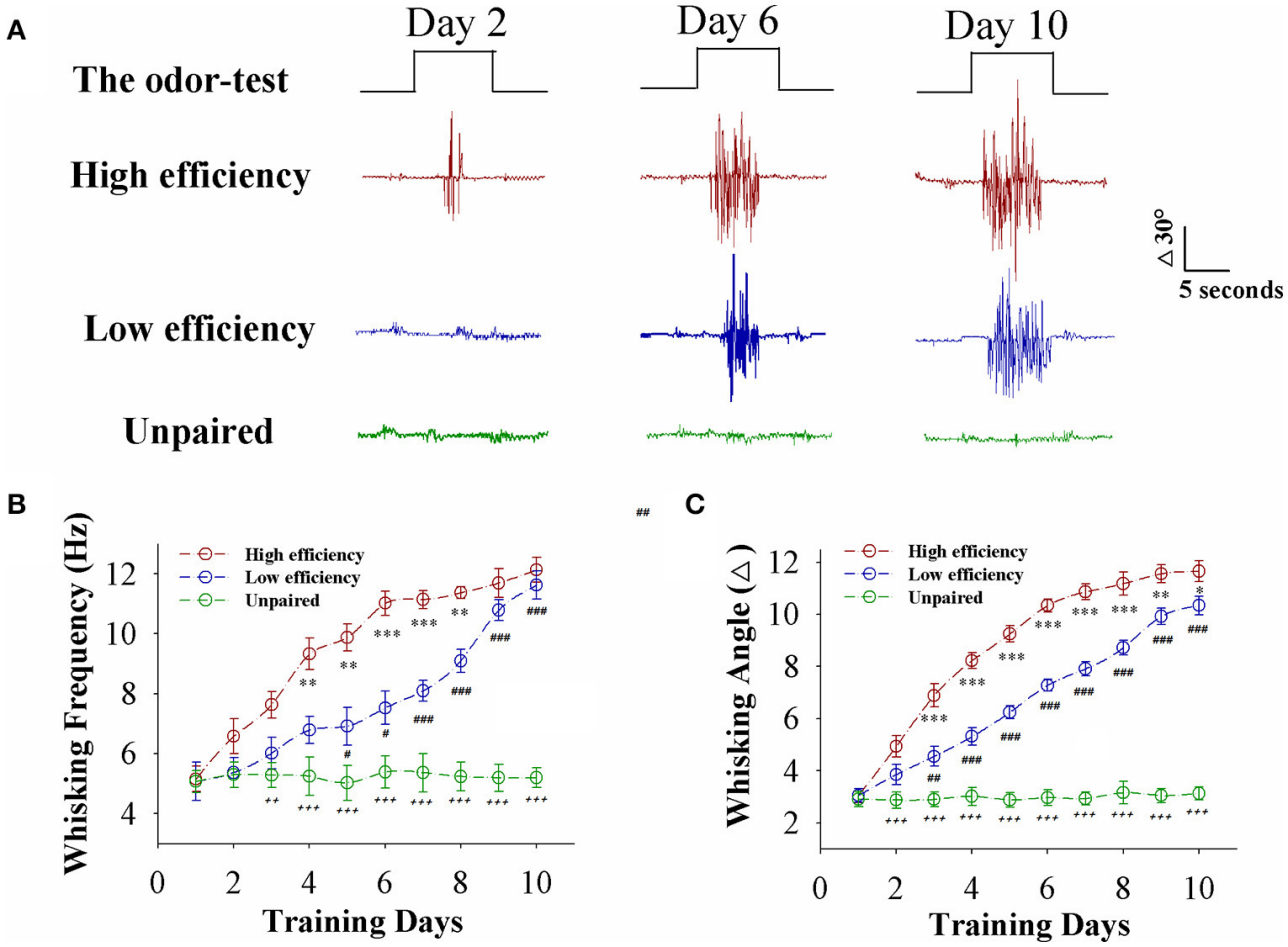
Paired whisker and odor stimulations lead to odorant-induced whisker motion in the forms of high and low efficiency. **(A)** Shows whisker motions in response to the odor-test pulses (black traces on top) in CR-formation mice with high efficiency (red traces) and low efficiency (blues) as well as an unpaired control mouse (greens) at training days 2 (left), 6 (middle), and 10 (right). Calibration bars are 30° and 5 s. **(B)** Shows whisking frequencies in response to the odor-test vs. training days in CR-formation mice with high efficiency (red symbols) and low efficiency (blue) as well as an unpaired control mice (green). (*n* = 9 mice for each group). **(C)** Shows whisking angles in response to the odor-test vs. training days in CR-formation mice with high efficiency (red symbols) and low efficiency (blue) as well as an unpaired control mice (green). (*n* = 9 mice for each group). Asterisks are used to show the comparison of groups between high and low learning efficiency. Pound signs are used to show the comparison of groups between low and unpaired control mice. Plus signs are used to show the comparison of groups between high and unpaired control mice. An asterisk, pound, or plus sign shows *p* < 0.05. Two asterisks, pound, or plus signs show *p* < 0.01. Three asterisks, pound, or plus signs show *p* < 0.001 (Statistical significance was determined using repeated ANOVA with a Bonferroni correction for multiple comparisons). t and DF values are given in Table S1.

Mice were divided into two groups that received the pairing of whisker stimulus (WS) and odor stimulus (OS) and the unpairing of WS and OS, respectively. The procedure consisted of each training for 20 s, five times in 2-h interval per day and 10 days (Wang et al., 2015; Gao et al., 2016; Yan et al., 2016). Odorant-induced whisker motion is established after pairing the WS and OS, compared to WS/OS unpair (Figure 1A). When analyzing the strength of odorant-induced whisker motion (a conditioned reflex, CR), we can see that the fully establishments of this associative memory need ~6 training days in certain mice (red trace) and 10 training days in others (blue), such that the establishment of this associative memory can be labeled as high efficiency and low efficiency, respectively (please see Methods for criteria). Figure 1B shows whisking frequency in response to the OS vs. training days from the mice with high efficiency (red symbols), low efficiency (blue), and unpaired control (green; *n* = 9 mice for each group). Figure 1C shows whisking angles in response to the OS vs. training days from the mice with high efficiency (red symbols), low efficiency (blue), and unpaired control (green; *n* = 9 mice for each group). Our data indicate that associative learning can be classified into high efficiency and low efficiency, similar to the natural process of learning and memory.

To study cellular mechanisms underlying the efficiency of associative learning, we have analyzed the plasticity in barrel cortical glutamatergic and GABAergic neurons from CR-formation mice with high and low learning efficiency.

### Barrel cortical glutamatergic neurons are upregulated with different efficiency after associative learning

Associative learning with high and low efficiency may be based on the differential strength of plasticity at glutamatergic neurons, which we have tested at YFP-labeled glutamatergic neurons in layers II~III of barrel cortices from unpaired control vs. CR-formation mice at training day six with high and low learning efficiency. In the coronal directions of brain slices that included barrel cortices, spontaneous excitatory postsynaptic currents (sEPSC) were recorded by whole-cell voltage clamp to assess excitatory synaptic activity. The ability to produce spikes was measured to estimate active intrinsic property under whole-cell current clamp. Spontaneous inhibitory postsynaptic currents (IPSC) were recorded by whole-cell voltage clamp to assess inhibitory synaptic function (Zhang et al., 2013; Gao et al., 2016).

**Figure 2:**
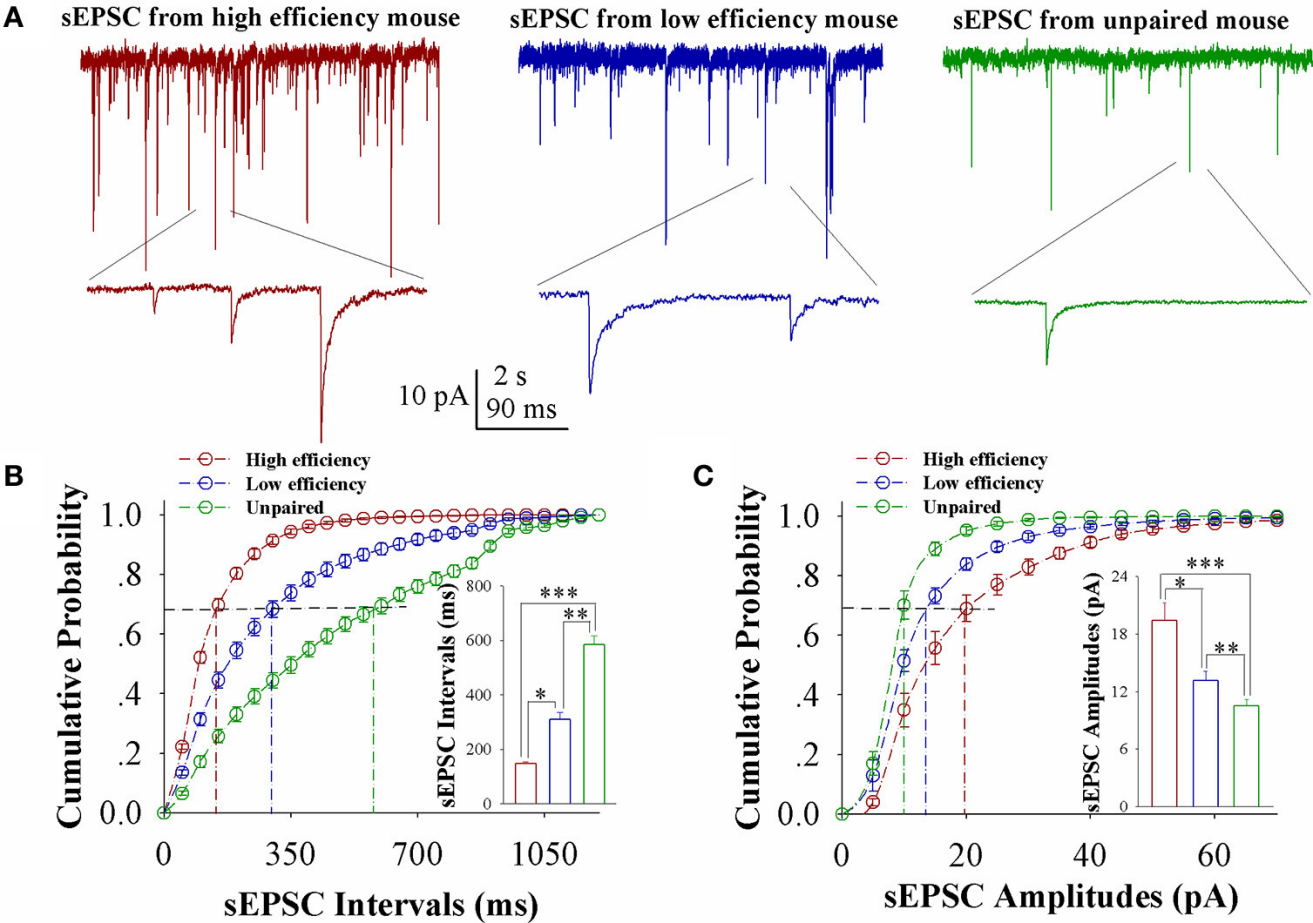
Excitatory synaptic transmission on barrel cortical glutamatergic neurons increases after pairing WS and OS, especially from the mice with the high efficiency of odorant-induced whisker motion. Spontaneous excitatory postsynaptic currents (sEPSC) were recorded on YFP-labeled glutamatergic neurons in cortical slices under voltage-clamp (holding potential at −70 mV) in presence of 10 μM bicuculline. **(A)** Shows sEPSCs recorded on the neurons from CR-formation mice with high learning efficiency (red traces) and low efficiency (blue) as well as from an unpaired control mouse (green). Bottom traces are the expanded waveforms selected from top traces. The calibration bars are 10 pA, 2 s (top) and 90 ms (bottom). **(B)** Illustrates cumulative probability vs. sEPSC intervals in the neurons from CR-formation mice with high learning efficiency (red symbols) and low efficiency (blue) as well as from unpaired control mice (green). Inserted figure shows the comparisons of sEPSC intervals at 67% cumulative probability from three groups of mice (*n* = 15 neurons from 9 mice for each group). **(C)** Illustrates cumulative probability vs. sEPSC amplitudes in the neurons from CR-formation mice with high efficiency (red symbols) and low efficiency (blue) as well as from unpaired control mice (green). Inserted figure denotes the comparisons of sEPSC amplitudes at 67% cumulative probability from three groups of mice (*n* = 15 neurons from 9 mice for each group). A one-way ANOVA with Bonferroni correction for multiple comparisons was performed to test for significant changes, one asterisk represents *p* < 0.05, two asterisks represent *p* < 0.01, and three asterisks represent *p* < 0.001. t and DF values are given in Table S2.

In comparisons of sEPSCs from CR-formation mice with high and low learning efficiency vs. unpaired controls, excitatory synaptic transmission on barrel cortical glutamatergic neurons appears to be increased in CR-formation mice, especially those with high learning efficiency (Figure 2A). Figure 2B shows cumulative probability vs. sEPSC intervals on glutamatergic neurons from the mice with high learning efficiency (red symbols), low efficiency (blue), and unpaired control (green; *n* = 15 neurons from 9 mice in each group). sEPSC intervals at 67% cumulative probability are 147.2 ± 6.63 ms on glutamatergic neurons from the mice with high learning efficiency (red bar in inserted figure), 309.73 ± 24.79 ms from the mice with low learning efficiency (blue) and 583.67 ± 32.28 ms in unpaired control mice (green), respectively (asterisk, *p* < 0.05; two asterisks, *p* < 0.01; and three asterisks, *p* < 0.001). Figure 2C illustrates cumulative probability vs. sEPSC amplitudes on glutamatergic neurons from the mice with high learning efficiency (red symbols), low efficiency (blue), and unpaired control (green; *n* = 15 neurons from 9 mice for each group). sEPSC amplitudes at 67% cumulative probability are 19.38 ± 1.86 pA on glutamatergic neurons from the mice with high learning efficiency (red bar in inserted figure), 13.18 ± 0.95 pA from the mice with low efficiency (blue), and 10.5 ± 0.66 pA from unpaired control mice (green), respectively (asterisk, *p* < 0.05; two asterisks, *p* < 0.01; and three asterisks, *p* < 0.001). Neuronal substrates for associative memory (odorant-induced whisker motion) may be based on the functional upregulation of the excitatory synaptic transmission on barrel cortical glutamatergic neurons and the strength of the upregulated synaptic transmission may be associated with learning efficiency.

**Figure 3:**
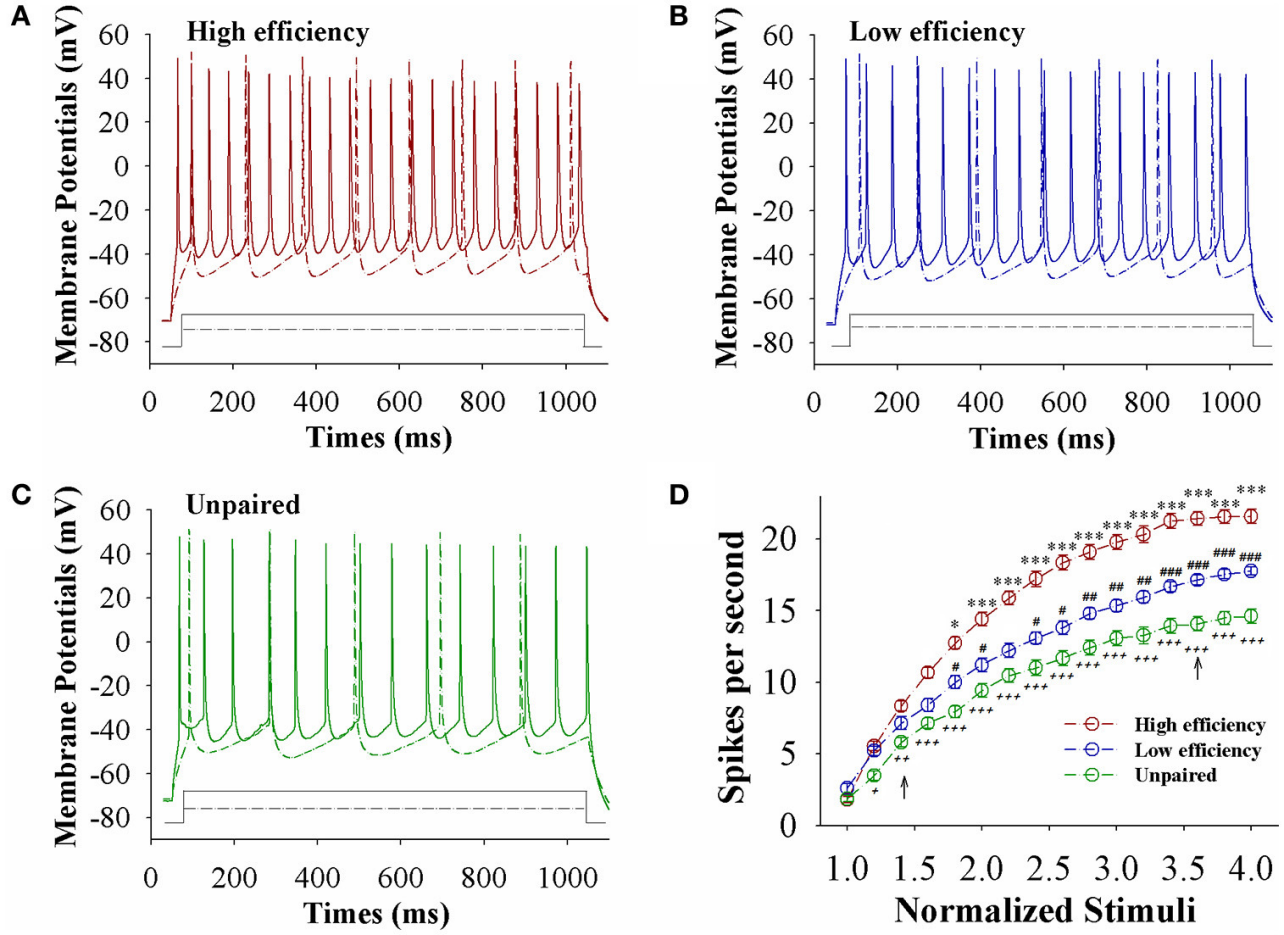
The capability to encode spikes on barrel cortical glutamatergic neurons increases after pairing WS and OS, especially in the mice with the high efficiency of odorant-induced whisker motion. Sequential spikes were induced by depolarization pulse under current-clamp recording on YFP-labeled glutamatergic neurons in cortical slices. **(A)** Shows the spikes induced two-steps of depolarization pulse on the neurons from a CR-formation mouse with high learning efficiency. **(B)** Shows the spikes induced two-steps of depolarization pulse on the neurons from a CR-formation mouse with low learning efficiency. **(C)** Shows the spikes induced two-steps of depolarization pulse on the neurons from an unpaired control mouse. **(D)** Shows spikes per second vs. normalized stimuli from control mice (green symbols) as well as CR-formation mice with high efficiency (red) and low efficiency (blue, *n* = 15 neurons from 9 mice for each group). Asterisks are used to show the comparison of groups between high and low learning efficiency. Pound signs are used to show the comparison of groups between low and unpaired control mice. Plus signs are used to show the comparison of groups between high and unpaired control mice. An asterisk, pound, or plus sign shows *p* < 0.05. Two asterisks, pound, or plus signs show *p* < 0.01. Three asterisks, pound, or plus signs show *p* < 0.001. Two-way ANOVA with Bonferroni correction for multiple comparisons was performed to test for significant changes. t and DF values are given in Table S3.

The capability of glutamatergic neurons to convert excitatory inputs into spikes appears upregulated in CR-formation mice (red and blue traces in Figures 3A–C), compared to those in unpaired control mice (green), especially in the mice with high learning efficiency (Figures 3A–C). Figure 3D illustrates spikes per second vs. normalized stimuli in barrel cortical glutamatergic neurons from CR-formation mice with high learning efficiency (red symbols) and low efficiency (blue) as well as in barrel cortical glutamatergic neurons from unpaired control mice (green), in which spikes per second are statistically different (*n* = 15 neurons from nine mice for each group, an asterisk, *p* < 0.05; two asterisks, *p* < 0.01; and three asterisks, *p* < 0.001). Associative learning upregulates the capability of barrel cortical glutamatergic neurons to convert excitatory inputs into digital spikes for information storage, especially in mice with high learning efficiency.

**Figure 4:**
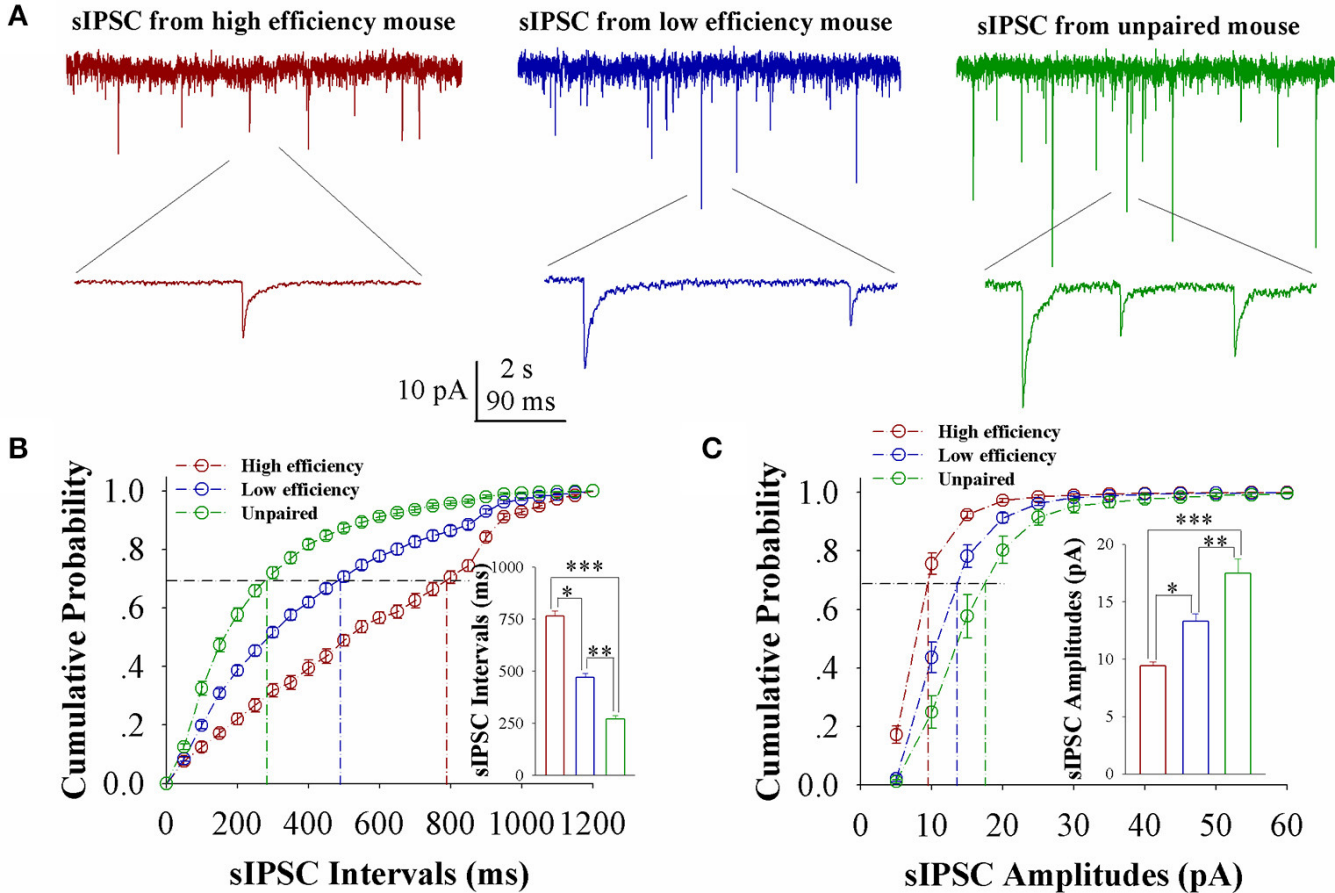
Inhibitory synaptic transmission on barrel cortical glutamatergic neurons decreases after pairing WS and OS, especially in the mice with the high efficiency of odorant-induced whisker motion. Spontaneous inhibitory postsynaptic currents (sIPSC) were recorded on YFP-labeled glutamatergic neurons in cortical slices under voltage-clamp (holding potential at −65 mV) in presence of 10 μM CNQX and 40 μM D-AP5. **(A)** Shows sIPSCs recorded on the neurons from CR-formation mice with high learning efficiency (red traces) and low efficiency (blue) as well as from an unpaired control mouse (green). Bottom traces are the expanded waveforms selected from top traces. The calibration bars are 10 pA, 2 s (top) and 90 ms (bottom). **(B)** Illustrates cumulative probability vs. sIPSC intervals in the neurons from CR-formation mice with high learning efficiency (red symbols) and low efficiency (blue) as well as from unpaired control mice (green). Inserted figure shows the comparisons of sIPSC intervals at 67% cumulative probability from three groups of mice (*n* = 15 neurons from 9 mice for each group). **(C)** Illustrates cumulative probability vs. sIPSC amplitudes in the neurons from CR-formation mice with high efficiency (red symbols) and low efficiency (blue) as well as from unpaired control mice (green). Inserted figure denotes the comparisons of sIPSC amplitudes at 67% cumulative probability from three groups of mice (*n* = 15 neurons from 9 mice for each group). A one-way ANOVA with Bonferroni correction for multiple comparisons was performed to test for significant changes, one asterisk represents *p* < 0.05, two asterisks represent *p* < 0.01, and three asterisks represent *p* < 0.001. t and DF values are given in Table S4.

The influence of associative learning on inhibitory synaptic transmission in barrel cortical glutamatergic neurons is showed in Figure 4. sIPSCs appears to be lowered in CR-formation mice, especially in those with high learning efficiency (Figure 4A). Figure 4B shows cumulative probability vs. sIPSC intervals on glutamatergic neurons from the mice with high learning efficiency (red symbols), low efficiency (blue), and unpaired control (green; *n* = 15 neurons from 9 mice in each group). sIPSC intervals at 67% cumulative probability are 765.23 ± 24.66 ms on glutamatergic neurons from the mice with high learning efficiency (red bar in inserted figure), 471.25 ± 17.86 ms from the mice with low learning efficiency (blue), and 270.92 ± 17.86 ms from unpaired control mice (green), respectively (asterisk, *p* < 0.05; two asterisks, *p* < 0.01; and three asterisks, *p* < 0.001). Figure 4C shows cumulative probability vs. sIPSC amplitudes on glutamatergic neurons from the mice with high learning efficiency (red symbols), low efficiency (blue), and unpaired control (green; *n* = 15 neurons from 9 mice for each group). sIPSC amplitudes at 67% cumulative probability are 9.43 ± 0.33 pA on glutamatergic neurons from the mice with high learning efficiency (red bar in inserted figure), 13.32 ± 0.65 pA from the mice with low learning efficiency (blue) and 17.48 ± 1.26 pA from unpaired control mice (green), respectively (asterisk, *p* < 0.05; two asterisks, *p* < 0.01; and three asterisks, *p* < 0.001). Neuronal substrates for associative memory may also be based on the functional downregulation of inhibitory synaptic transmission on barrel cortical glutamatergic neurons and the strength of the downregulated synaptic transmission may be associated with learning efficiency.

In summary, associative learning by pairing whisker and odor signals can lead to the upregulations of the excitatory synaptic transmission and the encoding capability as well as the downregulation of GABAergic synaptic transmission on glutamatergic neurons in the barrel cortex, especially under the condition of high learning efficiency. These changes may facilitate the recruitment and refinement of barrel cortical glutamatergic neurons as associative memory cells. We subsequently studied plasticity at barrel cortical inhibitory neurons after associative learning.

### Barrel cortical GABAergic neurons are downregulated with different efficiency after associative learning

In terms of plasticity at barrel cortical GABAergic neurons during associative learning, we have analyzed their excitatory synaptic inputs and ability to convert excitatory inputs into digital spikes at GFP-labeled GABAergic neurons in CR-formation mice and unpaired controls. sEPSCs were recorded to assess their receiving of excitatory synaptic transmission. The ability of converting excitatory inputs into digital spikes was measured to evaluate their active intrinsic properties (Zhang et al., 2013; Gao et al., 2016).

**Figure 5:**
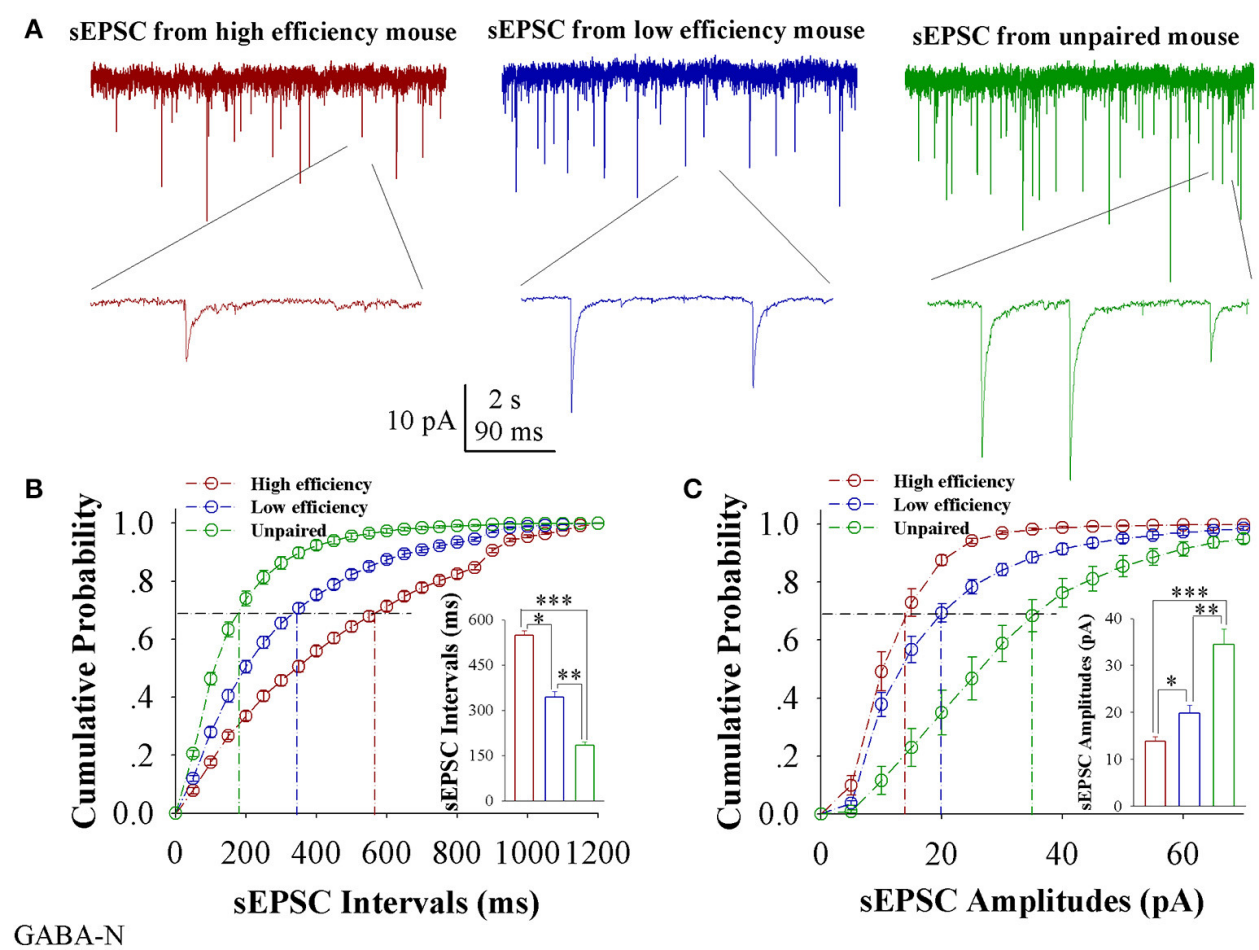
Excitatory synaptic transmission on barrel cortical GABAergic neurons decreases after pairing WS and OS, especially from the mice with the high efficiency of odorant-induced whisker motion. Spontaneous excitatory postsynaptic currents (sEPSC) were recorded on the GFP-labeled GABAergic neurons in cortical slices under voltage-clamp (holding potential at –65 mV) in presence of 10 μM bicuculline. **(A)** Shows sEPSCs recorded on the neurons from CR-formation mice with high learning efficiency (red traces) and low efficiency (blue) as well as from an unpaired control mouse (green). Bottom traces are the expanded waveforms selected from top traces. The calibration bars are 10 pA, 2 s (top) and 90 ms (bottom). **(B)** Illustrates cumulative probability vs. sEPSC intervals in the neurons from CR-formation mice with high learning efficiency (red symbols) and low efficiency (blue) as well as from unpaired control mice (green). Inserted figure shows the comparisons of sEPSC intervals at 67% cumulative probability from three groups of mice (*n* = 15 neurons from 9 mice for each group). **(C)** Illustrates cumulative probability vs. sEPSC amplitudes in the neurons from CR-formation mice with high efficiency (red symbols) and low efficiency (blue) as well as from unpaired control mice (green). Inserted figure denotes the comparisons of sIPSC amplitudes at 67% cumulative probability from three groups of mice (*n* = 15 neurons from 9 mice for each group). A one-way ANOVA with Bonferroni correction for multiple comparisons was performed to test for significant changes, one asterisk represents *p* < 0.05, two asterisks represent *p* < 0.01, and three asterisks represent *p* < 0.001. t and DF values are given in Table S5.

In comparisons of sEPSCs from CR-formation mice with high and low learning efficiency vs. unpaired controls, excitatory synaptic transmission on barrel cortical GABAergic neurons appears decreased in CR-formation mice, especially those with high learning efficiency (Figure 5A). Figure 5B illustrates cumulative probability vs. sEPSC intervals on GABAergic neurons from the mice with high learning efficiency (red symbols), low efficiency (blue), and unpaired control (green; *n* = 15 neurons from 9 mice in each group). sEPSC intervals at 67% cumulative probability are 547.73 ± 15.98 ms on GABAergic neurons from the mice with high learning efficiency (red bar in inserted figure), 342.13 ± 18.82 ms from the mice with low efficiency (blue), and 184.2 ± 10.39 ms in unpaired control mice (green), respectively (asterisk, *p* < 0.05; two asterisks, *p* < 0.01; and three asterisks, *p* < 0.001). Figure 5C shows cumulative probability vs. sEPSC amplitudes on GABAergic neurons from the mice with high learning efficiency (red symbols), low efficiency (blue), and unpaired control (green; *n* = 15 neurons from 9 mice for each group). sEPSC amplitudes at 67% cumulative probability are 13.83 ± 1.01 pA on GABAergic neurons from the mice with high learning efficiency (red bar in inserted figure), 19.75 ± 1.83 pA from the mice with low efficiency (blue) and 34.39 ± 3.38 pA from unpaired control mice (green), respectively (asterisk, *p* < 0.05; two asterisks, *p* < 0.01; and three asterisks, *p* < 0.001). Therefore, neuronal substrates for associative memory (odorant-induced whisker motion) may be based on the functional downregulation of excitatory synaptic transmission on barrel cortical GABAergic neurons and the strength of the downregulated synaptic transmission may be associated with learning efficiency.

**Figure 6:**
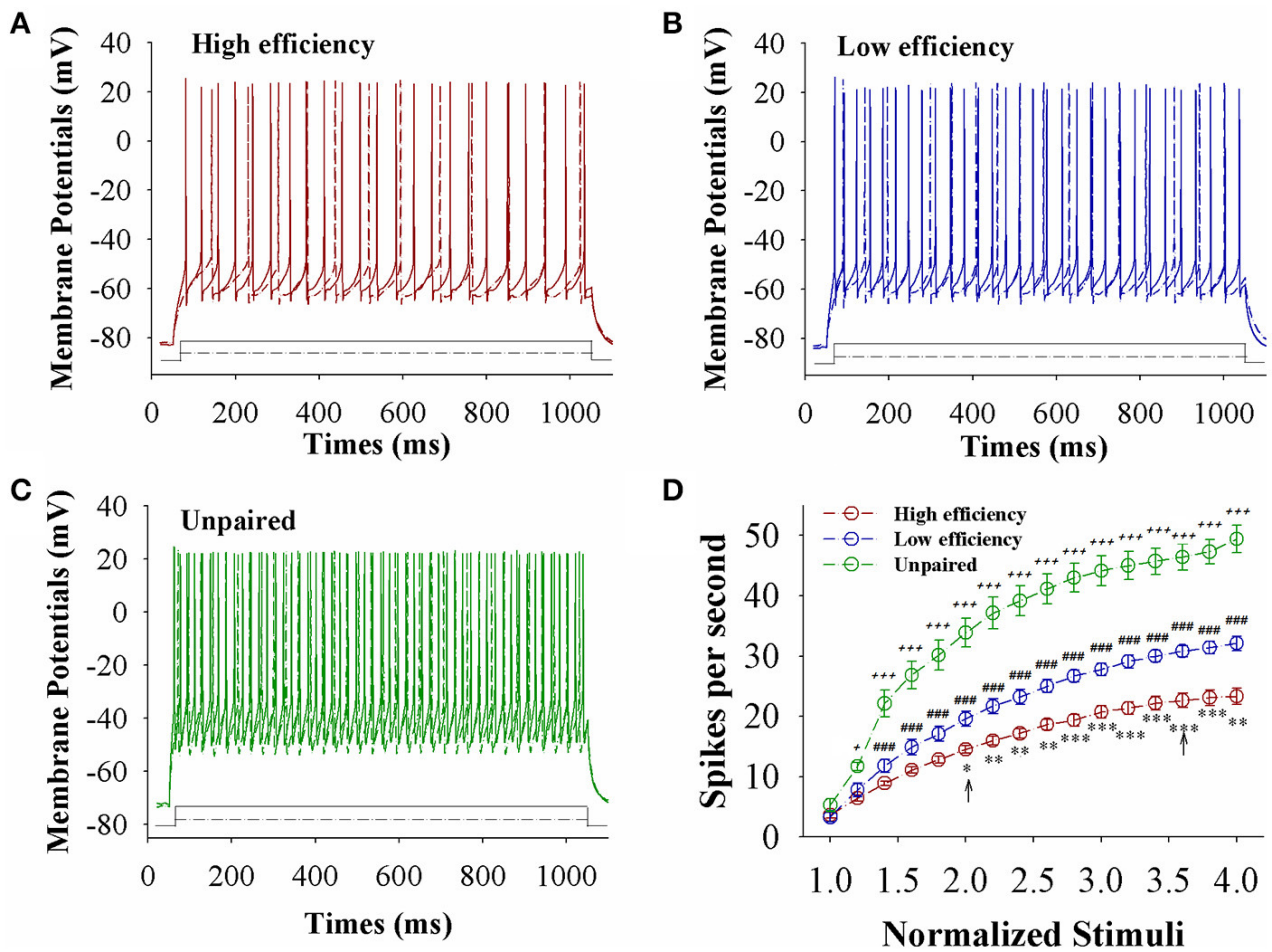
The capability to encode spikes on barrel cortical GABAergic neurons decreases after pairing WS and OS, especially in the mice with the high efficiency of odorant-induced whisker motion. Sequential spikes were induced by Depolarization pulse under current-clamp recording on GFP-labeled GABAergic neurons in cortical slices. **(A)** Shows the spikes induced two-steps of depolarization pulse on the neurons from a CR-formation mouse with high learning efficiency. **(B)** Illustrates the spikes induced two-steps of depolarization pulse on the neurons from a CR-formation mouse with low learning efficiency. **(C)** Shows the spikes induced two-steps of depolarization pulse on the neurons from an unpaired control mouse. **(D)** Shows spikes per second vs. normalized stimuli from control mice (green symbols) as well as CR-formation mice with high efficiency (red) and low efficiency (blue, *n* = 11 neurons from 9 mice for each group). Asterisks are used to show the comparison of groups between high and low learning efficiency. Pound signs are used to show the comparison of groups between low and unpaired control mice. Plus signs are used to show the comparison of groups between high and unpaired control mice. An asterisk, pound or plus sign shows *p* < 0.05. Two asterisks, pound, or plus signs show *p* < 0.01. Three asterisks, pound, or plus signs show *p* < 0.001. Two-way ANOVA with Bonferroni correction for multiple comparisons was performed to test for significant changes. t and DF values are given in Table S6.

The capability of GABAergic neurons to convert excitatory inputs into spikes appears downregulated in CR-formation mice (red and blue traces), compared with those in unpaired control mice (green), especially in the mice with high learning efficiency (Figures 6A–C). Figure 6D illustrates spikes per second vs. normalized stimuli in barrel cortical glutamatergic neurons from CR-formation mice with high learning efficiency (red symbols) and low efficiency (blue) as well as in barrel cortical glutamatergic neurons from unpaired control mice (green), where spikes per second are statistically different (*n* = 11 neurons from nine mice for each group, asterisk, *p* < 0.05; two asterisks, *p* < 0.01; and three asterisks, *p* < 0.001). Associative learning downregulates the capability of barrel cortical GABAergic neurons to convert excitatory inputs into digital spikes for information storage, especially in mice with high learning efficiency.

In summary, associative learning downregulates excitatory synaptic inputs and spike-encoding capability in barrel cortical GABAergic neurons, especially under the condition of high learning efficiency. The downregulations of the encoding ability in GABAergic neurons and their output synapse functions may facilitate the recruitment and refinement of glutamatergic neurons in the barrel cortex after associative learning.

### Neural plasticity strength is correlated with associative learning efficiency

**Figure 7:**
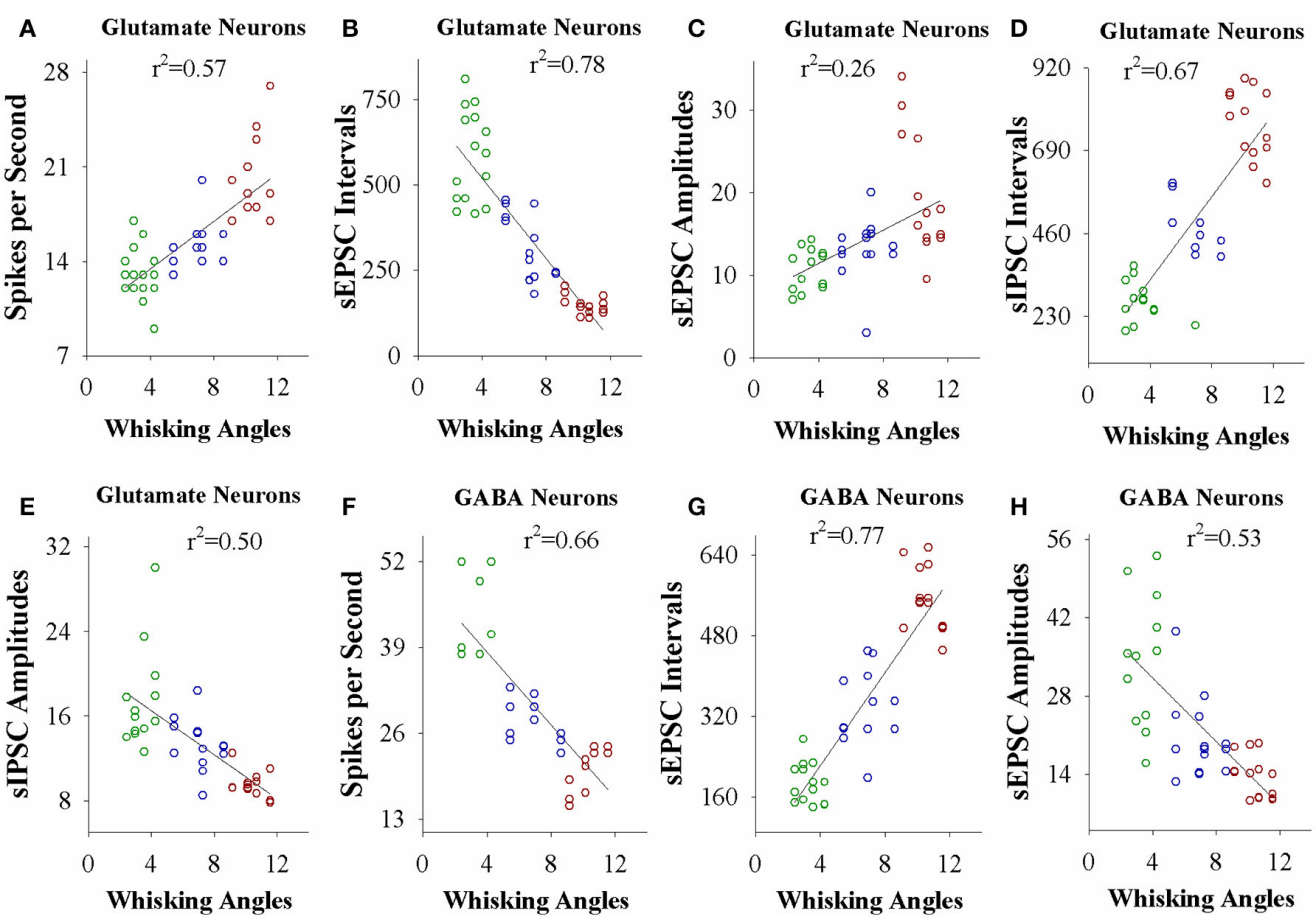
The activity strength of barrel cortical glutamatergic and GABAergic neurons is linearly correlated to the efficiency of odorant-induced whisker motion in whisking angles from individual neurons vs. correspondent mice. **(A)** Shows a correlation between spike per second on glutamatergic neurons and whisking angles induced by the odor-test. **(B)** Illustrates a correlation between sEPSC intervals on glutamatergic neurons and whisking angles. **(C)** Shows a correlation between sEPSC amplitude on glutamatergic neurons and whisking angles. **(D)** Illustrates a correlation between sIPSC intervals on glutamatergic neurons and whisking angles. **(E)** Shows a correlation between sIPSC amplitudes on glutamatergic neurons and whisking angles. **(F)** Illustrates a correlation between spike per second on GABAergic neurons and whisking angles. **(G)** Illustrates a correlation between sEPSC intervals on GABAergic neurons and whisking angles. **(H)** Illustrates a correlation between sEPSC amplitudes on GABAergic neurons and whisking angles.

**Figure 8:**
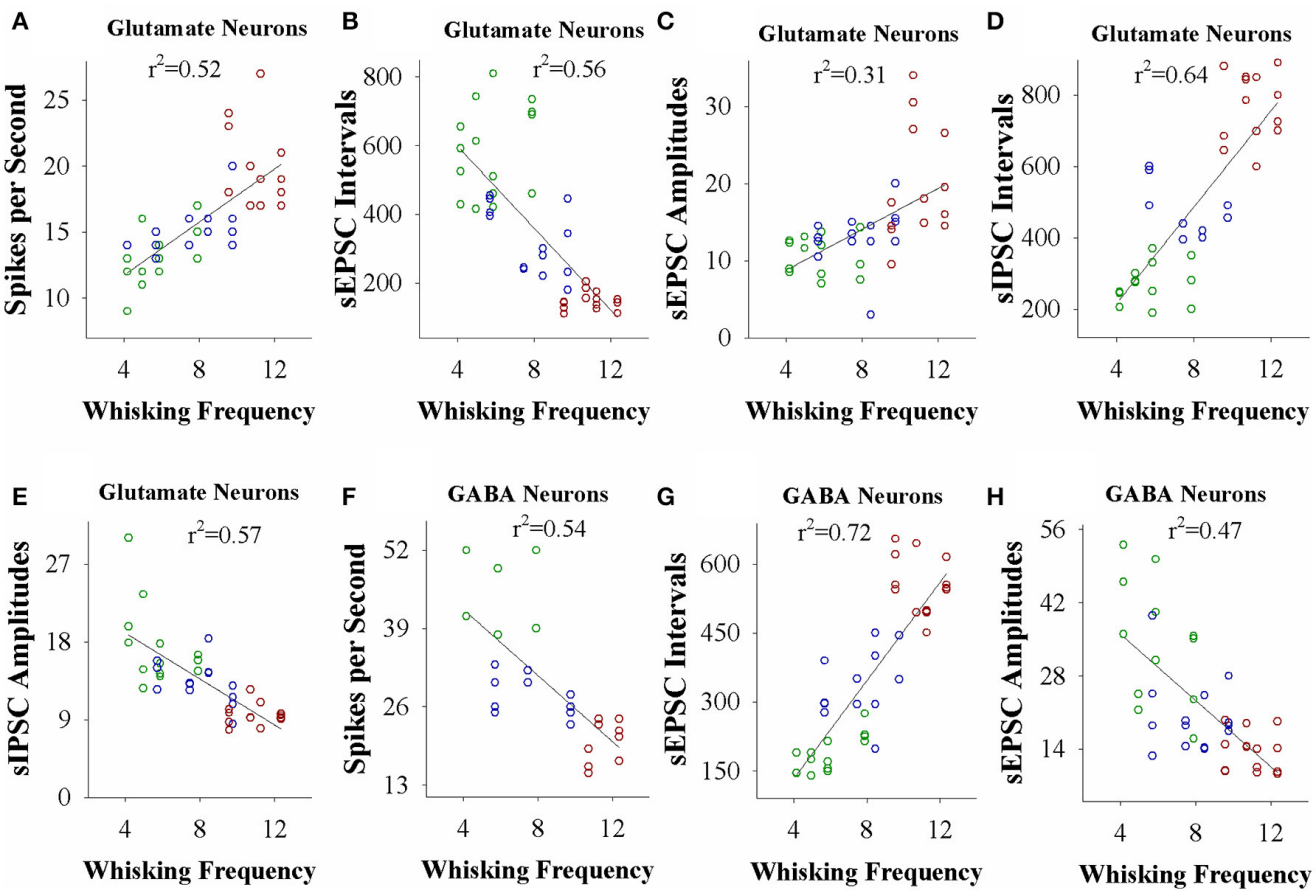
The activity strength of barrel cortical glutamatergic and GABAergic neurons is linearly correlated to the efficiency of odorant-induced whisker motion in whisking frequency from individual neurons vs. correspondent mice. **(A)** Shows a correlation between spike per second on glutamatergic neurons and whisking frequency induced by the odor-test. **(B)** Illustrates a correlation between sEPSC intervals on glutamatergic neurons and whisking frequency. **(C)** Shows a correlation between sEPSC amplitudes on glutamatergic neurons and whisking frequency. **(D)** Shows a correlation between sIPSC intervals on glutamatergic neurons and whisking frequency. **(E)** Illustrates a correlation between sIPSC amplitudes on glutamatergic neurons and whisking frequency. **(F)** Illustrates a correlation between spike per second on GABAergic neurons and whisking frequency. **(G)** Illustrates a correlation between sEPSC intervals on GABAergic neurons and whisking frequency. **(H)** Illustrates a correlation between sEPSC amplitudes on GABAergic neurons and whisking frequency.

If the strength of neuronal plasticity is correlated to the efficiency of cross-modal associative memory, the establishment of associative memory is set by neuronal plasticity. To examine this hypothesis, we took the following parameters into our analysis. The strengths of associative memory, such as the angles and frequencies of the whisker fluctuation in response to the odor-test, in CR-formation mice with high and low efficiencies at training day 6 as well as UPS mice were plotted in X-axis. The amplitudes and intervals of sEPSCs and sIPSCs at 67% cumulative probability as well as the number of spikes induced by 3.0 normalized stimuli in input-output curves were plotted in Y-axis. As illustrated in Figures 7, 8, the strengths of associative memory are linearly correlated to synaptic efficacy and spiking ability in the barrel cortices. Therefore, cellular mechanism underlying associative memory is correlated to neuronal plasticity in terms of the upregulation of excitatory synaptic transmission and spike ability as well as the downregulation of inhibitory synaptic transmission on the glutamatergic neurons.

## Discussion

In the mice that show odorant-induced whisker motion, its fully establishment appears to be fast and slow, i.e., the formation of associative memory can be classified into high learning efficiency and low learning efficiency (Figure 1). Barrel cortical glutamatergic neurons express the increases in excitatory synaptic transmission and spiking ability as well as the decreases in inhibitory synaptic transmission (Figures 2–4). In the meantime, barrel cortical GABAergic neurons express the decreases in their excitatory synaptic transmission and spiking ability (Figures 5, 6). Furthermore, the strengths in the upregulation of glutamatergic neurons and the downregulation of GABAergic neurons are more dominant in the mice with high learning efficiency than in those with low learning efficiency, i.e., the strength of neuronal plasticity is linearly correlated with the efficiency of associative learning memory (Figures 7, 8). Coordinated refinements in the upregulation of glutamatergic neurons and the downregulation of GABAergic neurons may facilitate their recruitments to be associative memory cells and drive them to optimal state information storages and retrievals (Yan et al., 2016). Compared with our previous studies (Wang et al., 2015; Gao et al., 2016; Yan et al., 2016; Guo et al., 2017), the present study reveals that associative learning and memory can be classified into high efficiency and low efficiency, with which the strength of neuronal plasticity is linearly correlated. Our study through quantitative analyses of correlations between associative neuronal plasticity and learning efficiency further supports the belief that neural plasticity is associated with learning and memory in juvenile mice.

A common sense is that the ability of learning and memory is variable among the individuals and even in the same individual under different conditions. If memory cells' recruitment and their functional plasticity are cellular mechanisms for information storage (Wang et al., 2015; Gao et al., 2016; Yan et al., 2016; Guo et al., 2017; Wang and Cui, 2017), the strength of neuronal plasticity and memory cell recruitment should be comparable with the efficiency of learning and memory. In this study, we present that the levels of associative learning and memory are parallel to the plasticity strengths of sensory cortical glutamatergic and GABAergic neurons (Figures 7, 8). Moreover, the differences between memory strength and associative memory cells are comparable in ipsilateral and contralateral sides (Gao et al., 2016). Although these data support the hypotheses above, the relationship between memory cell recruitment and learning efficiency as well as the influence of various learning conditions on memory efficiency remain to be studied.

To the roles of plasticity at barrel cortical glutamatergic and GABAergic neurons in associative memory, we propose that the upregulation of excitatory neurons by increasing glutamatergic synaptic transmission and their excitability as well as the downregulation of inhibitory neuros by decreasing GABAergic synaptic transmission and their excitability make these glutamatergic neurons to be more excitable, which may permit the excitatory driving force from the new synapse innervation of the piriform cortex to recruit them as associative memory cells (Wang et al., 2015; Gao et al., 2016; Wang J.-H. et al., 2016; Yan et al., 2016; Guo et al., 2017) and to refine them with the upregulated ability to encode digital spikes (Wang et al., 2008; Yu et al., 2011, 2012) for information storage. In terms of information retrieval, associative memory cells, and their upregulations boost their capability to activate the neurons in downstream brain areas for behavioral reaction and memory presentation. If the sensitivity and intrinsic property of these associative memory cells are upregulated, their strong driving force to downstream neurons may lead to the elevated activities in the brain areas related to behavior and emotion reactions, and the pathological associations may be related to illusion, delusion, and convulsion (Wang and Cui, 2017).

The upregulation of glutamatergic synapses and neurons as well as the downregulation of GABAergic synapses and neurons may facilitate the recruitment of associative memory cells and boost these memory cells to an optimal state for information storage (Yan et al., 2016). Our current study further indicates the linear correlation between these regulations and learning efficiency, such that the hypothesis about the functional impact of their coordination is strengthened. It remains to be investigated why glutamatergic synaptic transmission and neuronal excitability are upregulated and GABAergic synaptic transmission and neuronal excitability are downregulated coordinately. We have proposed intracellular signaling pathways may coordinate these changes since Ca^2+^ signal coordinates the functions of different subcellular compartments (Chen et al., 2008), which we are studying.

Taken the current data with our previous studies (Wang et al., 2015; Gao et al., 2016; Wang J.-H. et al., 2016; Yan et al., 2016; Guo et al., 2017) together, we propose that the efficiency of associative learning is proportional to the recruitment of associative memory cells and the strength of their functional plasticity, and furthermore, the portion of associative memory cells and the strength of their plasticity will be proportional to the power of information storage, which are indicated by our current studies (Gao et al., 2016; Guo et al., 2017). In this regard, learning efficiency and memory strength can be considered to be similar meaning in entire processes of learning and memory. The differences in learning efficiency and memory strength in the mice are due to the differences in the number and functional plasticity of associative memory cells.

It is noteworthy that in our previous study, we did not pay attention to the difference of learning efficiency, i.e., we trained the mice up to day 10 for them to have odorant-induced whisker motion reaching to plateau level, and subsequently conducted the study of cellular mechanisms by two-photon cell imaging and electrophysiology. Although learning efficiency by measuring the time of odorant-induced whisker motion to the plateau level is different, cellular mechanisms after learning efficiency and memory strength reach to the plateau level may be similar for the groups of mice with high and low efficiencies. This point is supported by linear correlation between learning efficiency and neuronal plasticity. Moreover, the data in present study are not from those in previous studies, in which different researchers are involved from different Institutions although senior author is identical. The similar results from different researchers, Institutions and locations make our studies convincing.

In terms of the recruitment of associative memory cells, the coordinated plasticity of these cells as well as the parallel relationship between learning efficiency and neural plasticity, we propose the following molecular and cellular processes. The associated activations of the barrel and piriform cortices induce epigenetic-mediated changes (Yan et al., 2016). The upregulated miRNAs knock down their target genes, or vice versa. The altered expression of the genes and proteins facilitates axon prolongation, new synapse innervation and excitatory synapse function, as well as attenuates inhibitory synapse function. These changes lead to the coordinated recruitment and refinement of glutamatergic and GABAergic neurons to be associative memory cells. This assumption is supported by our current observation that anti-miRNA-324 and anti-miRNA-133a block associative memory and synapse innervation (Wang J.-H. et al., 2016; Wang et al., 2017).

## Author contributions

JW contributed project design and paper writing. XZ, LH, YL, RGu, SZ, SG, RGe, SC, and SW contributed experiments and data analyses.

### Conflict of interest statement

The authors declare that the research was conducted in the absence of any commercial or financial relationships that could be construed as a potential conflict of interest.

## References

[B1] Ascoli G. A. Alonso-Nanclares L. Anderson S. A. Barrionuevo G. Benavides-Piccione R. Burkhalter A.. (2008). Petilla terminology: nomenclature of features of GABAergic interneurons of the cerebral cortex. Nat. Rev. Neurosci. 9, 557–568. 10.1038/nrn2402 18568015 PMC2868386

[B2] Bienvenu T. C. Busti D. Magill P. J. Ferraguti F. Capogna M. (2012). Cell-type-specific recruitment of amygdala interneurons to hippocampal theta rhythm and noxious stimuli *in vivo*. Neuron 74, 1059–1074. 10.1016/j.neuron.2012.04.022 22726836 PMC3391683

[B3] Blair H. T. Schafe G. E. Bauer E. P. Rodrigues S. M. LeDoux J. E. (2001). Synaptic plasticity in the lateral amygdala: a cellular hypothesis of fear conditioning. Learn. Mem. 8, 229–242. 10.1101/lm.30901 11584069

[B4] Cai D. J. Aharoni D. Shuman T. Shobe J. Biane J. Song W.. (2016). A shared neural ensemble links distinct contextual memories encoded close in time. Nature 534, 115–118. 10.1038/nature17955 27251287 PMC5063500

[B5] Cheetham C. E. Barnes S. J. Albieri G. Knott G. W. Finnerty G. T. (2012). Pansynaptic enlargement at adult cortical connections strengthened by experience. Cereb. Cortex 24, 521–531. 10.1093/cercor/bhs334 23118196 PMC3888373

[B6] Chen N. Chen X. Wang J.-H. (2008). Homeostasis established by coordination of subcellular compartment plasticity improves spike encoding. J. Cell Sci. 121, 2961–2971. 10.1242/jcs.022368 18697837

[B7] Chen N. Chen X. Yu J. Wang J.-H. (2006a). After-hyperpolarization improves spike programming through lowering threshold potentials and refractory periods mediated by voltage-gated sodium channels. Biochem. Biophys. Res. Commun. 346, 938–945. 10.1016/j.bbrc.2006.06.003 16777065

[B8] Chen N. Zhu Y. Gao X. Guan S. Wang J.-H. (2006b). Sodium channel-mediated intrinsic mechanisms underlying the differences of spike programming among GABAergic neurons. Biochem. Biophys. Res. Commun. 346, 281–287. 10.1016/j.bbrc.2006.05.120 16756951

[B9] Christian K. M. Thompson R. F. (2003). Neural substrates of eyeblink conditioning: acquisition and retention. Learn. Mem. 10, 427–455. 10.1101/lm.59603 14657256

[B10] DeFelipe J. Lopez-Cruz P. L. Benavides-Piccione R. Bielza C. Larranaga P. Anderson S.. (2013). New insights into the classification and nomenclature of cortical GABAergic interneurons. Nat. Rev. Neurosci. 14, 202–216. 10.1038/nrn3444 23385869 PMC3619199

[B11] Dityatev A. E. Bolshakov V. Y. (2005). Amygdala, long-term potentiation, and fear conditioning. Neuroscientist 11, 75–88. 10.1177/1073858404270857 15632280

[B12] Fanselow M. S. Poulos A. M. (2005). The neuroscience of mammalian associative learning. Annu. Rev. Psychol. 56, 207–234. 10.1146/annurev.psych.56.091103.070213 15709934

[B13] Frey S. Frey J. U. (2008). ‘Synaptic tagging’ and ‘cross-tagging’ and related associative reinforcement processes of functional plasticity as the cellular basis for memory formation. Prog. Brain Res. 169, 117–143. 10.1016/S0079-6123(07)00007-6 18394471

[B14] Gao Z. Chen L. Fan R. Lu W. Wang D. Cui S.. (2016). Associations of unilateral whisker and olfactory signals induce synapse formation and memory cell recruitment in bilateral barrel cortices: cellular mechanism for unilateral training toward bilateral memory. Front. Cell. Neurosci. 10:285. 10.3389/fncel.2016.00285 28018178 PMC5160353

[B15] Ge R. Qian H. Chen N. Wang J. H. (2014). Input-dependent subcellular localization of spike initiation between soma and axon at cortical pyramidal neurons. Mol. Brain 7:26. 10.1186/1756-6606-7-26 24708847 PMC4022375

[B16] Ge R. Qian H. Wang J. H. (2011). Physiological synaptic signals initiate sequential spikes at soma of cortical pyramidal neurons. Mol. Brain 4:19. 10.1186/1756-6606-4-19 21549002 PMC3113741

[B17] Guo R. Ge R. Zhao S. Liu Y. Zhao X. Huang L.. (2017). Associative memory extinction is accompanied by decayed plasticity at motor cortical neurons and persistent plasticity at sensory cortical neurons. Front. Cell. Neurosci. 11:168. 10.3389/fncel.2017.00168 28659764 PMC5469894

[B18] Harlow E. G. Till S. M. Russell T. A. Wijetunge L. S. Kind P. Contractor A. (2010). Critical period plasticity is disrupted in the barrel cortex of FMR1 knockout mice. Neuron 65, 385–398. 10.1016/j.neuron.2010.01.024 20159451 PMC2825250

[B19] Honey R. C. Good M. (2000). Associative components of recognition memory. Curr. Opin. Neurobiol. 10, 200–204. 10.1016/S0959-4388(00)00069-6 10753791

[B20] Jones N. G. Kemenes I. Kemenes G. Benjamin P. R. (2003). A persistent cellular change in a single modulatory neuron contributes to associative long-term memory. Curr. Biol. 13, 1064–1069. 10.1016/S0960-9822(03)00380-4 12814554

[B21] Letzkus J. J. Wolff S. B. Meyer E. M. Tovote P. Courtin J. Herry C.. (2012). A disinhibitory microcircuit for associative fear learning in the auditory cortex. Nature 480, 331–335. 10.1038/nature10674 22158104

[B22] Lu W. Wen B. Zhang F. Wang J. H. (2014). Voltage-independent sodium channels emerge for an expression of activity-induced spontaneous spikes in GABAergic neurons. Mol. Brain 7:38. 10.1186/1756-6606-7-38 24886791 PMC4039334

[B23] Ma K. Guo L. Xu A. Cui S. Wang J. H. (2016a). Molecular Mechanism for stress-induced depression assessed by sequencing miRNA and mRNA in medial prefrontal cortex. PLoS ONE 11:e0159093. 10.1371/journal.pone.0159093 27427907 PMC4948880

[B24] Ma K. Xu A. Cui S. Sun M. Xue Y. Wang J.-H. (2016b). Impaired GABA synthesis, uptake and release are associated with depression-like behaviors induced by chronic mild stress. Transl. Psychiatry 6, 1–10. 10.1038/tp.2016.181 27701406 PMC5315548

[B25] Margolis D. J. Lutcke H. Schulz K. Haiss F. Weber B. Kugler S.. (2012). Reorganization of cortical population activity imaged throughout long-term sensory deprivation. Nat. Neurosci. 15, 1539–1546. 10.1038/nn.3240 23086335

[B26] McKay B. E. Turner R. W. (2005). Physiological and morphological development of the rat cerebellar purkinje cell. J. Physiol. 567, 829–850. 10.1113/jphysiol.2005.089383 16002452 PMC1474219

[B27] Neves G. Cooke S. F. Bliss T. V. (2008). Synaptic plasticity, memory and the hippocampus: a neural network approach to causality. Nat. Rev. Neurosci. 9, 65–75. 10.1038/nrn2303 18094707

[B28] Ni H. Huang L. Chen N. Zhang F. Liu D. Ge M.. (2010). Upregulation of barrel GABAergic neurons is associated with cross-modal plasticity in olfactory deficit. PLoS ONE 5:e13736. 10.1371/journal.pone.0013736 21060832 PMC2966404

[B29] Nikitin E. S. Vavoulis D. V. Kemenes I. Marra V. Pirger Z. Michel M.. (2008). Persistent sodium current is a nonsynaptic substrate for long-term associative memory. Curr. Biol. 18, 1221–1226. 10.1016/j.cub.2008.07.030 18701288

[B30] Rosselet C. Fieschi M. Hugues S. Bureau I. (2011). Associative learning changes the organization of functional excitatory circuits targeting the supragranular layers of mouse barrel cortex. Front. Neural Circuits 4:126. 10.3389/fncir.2010.00126 21267427 PMC3024829

[B31] Silva A. J. (2003). Molecular and cellular cognitive studies of the role of synaptic plasticity in memory. J. Neurobiol. 54, 224–237. 10.1002/neu.10169 12486706

[B32] Stevens C. F. (2004). Presynaptic function. Curr. Opin. Neurobiol. 14, 341–345. 10.1016/j.conb.2004.04.004 15194114

[B33] Suzuki W. A. (2008). Associative learning signals in the brain. Prog. Brain Res. 169, 305–320. 10.1016/S0079-6123(07)00019-2 18394483

[B34] Takehara-Nishiuchi K. McNaughton B. L. (2008). Spontaneous changes of neocortical code for associative memory during consolidation. Science 322, 960–963. 10.1126/science.1161299 18988855

[B35] Vincis R. Fontanini A. (2016). Associative learning changes cross-modal representations in the gustatory cortex. Elife 5:e16420. 10.7554/eLife.16420 27572258 PMC5026467

[B36] Viskontas I. V. (2008). Advances in memory research: single-neuron recordings from the human medial temporal lobe aid our understanding of declarative memory. Curr. Opin. Neurol. 21, 662–668. 10.1097/WCO.0b013e3283168e03 18989110

[B37] Wang D. Zhao J. Gao Z. Chen N. Wen B. Lu W.. (2015). Neurons in the barrel cortex turn into processing whisker and odor signals: a cellular mechanism for the storage and retrieval of associative signals. Front. Cell. Neurosci. 9:320. 10.3389/fncel.2015.00320 26347609 PMC4543922

[B38] Wang G. Y. Zhu Z. M. Cui S. Wang J. H. (2016). Glucocorticoid induces incoordination between glutamatergic and GABAergic neurons in the amygdala. PLoS ONE 11:e0166535. 10.1371/journal.pone.0166535 27861545 PMC5115758

[B39] Wang J.-H. (2003). Short-term cerebral ischemia causes the dysfunction of interneurons and more excitation of pyramidal neurons. Brain Res. Bull. 60, 53–58. 10.1016/S0361-9230(03)00026-1 12725892

[B40] Wang J. H. Chen N. Gao Z. L. Wen B. Yan F. X. Chen P. (2014). Upregulation of glutamatergic receptor-channels is associated with cross-modal reflexes encoded in barrel cortex and piriform cortex. Biophys. J. 106(Suppl. 191a). 10.1016/j.bpj.2013.11.1114

[B41] Wang J.-H. Cui S. (2017). Associative memory cells: formation, function and perspective. F1000Res. 6, 283. 10.12688/f1000research.11096.2 28408978 PMC5373424

[B42] Wang J.-H. Feng J. Lu W. (2017). Associative memory cells are recruited to encode triple sensory signals via synapse formation. Biophys. J. 112(Suppl. 1), 443a–444a. 10.1016/j.bpj.2016.11.2377

[B43] Wang J.-H. Kelly P. T. (2001). Ca2^+^/CaM signalling pathway up-regulates glutamatergic synaptic function in non-pyramidal fast-spiking neurons of hippocampal CA1. J. Physiol. 533, 407–422. 10.1111/j.1469-7793.2001.0407a.x 11389201 PMC2278630

[B44] Wang J.-H. Wang D. Gao Z. Chen N. Lei Z. Cui S. (2016). Both glutamatergic and gabaergic neurons are recruited to be associative memory cells. Biophys. J. 110(suppl.481a). 10.1016/j.bpj.2015.11.2571

[B45] Wang J. H. Wei J. Chen X. Yu J. Chen N. Shi J. (2008). The gain and fidelity of transmission patterns at cortical excitatory unitary synapses improve spike encoding. J. Cell Sci. 121, 2951–2960. 10.1242/jcs.025684 18697836

[B46] Wasserman E. A. Miller R. R. (1997). What's elementary aboutassociative learning? Annu. Rev. Psychol. 48, 573–607. 10.1146/annurev.psych.48.1.573 9046569

[B47] Weeks A. C. Connor S. Hinchcliff R. LeBoutillier J. C. Thompson R. F. Petit T. L. (2007). Eye-blink conditioning is associated with changes in synaptic ultrastructure in the rabbit interpositus nuclei. Learn. Mem. 14, 385–389. 10.1101/lm.348307 17551096 PMC1896088

[B48] Wei J. Zhang M. Zhu Y. Wang J. H. (2004). Ca2^+^-calmodulin signalling pathway upregulates GABA synaptic transmission through cytoskeleton-mediated mechanisms. Neuroscience 127, 637–647. 10.1016/j.neuroscience.2004.05.056 15283963

[B49] Wesson D. W. Donahou T. N. Johnson M. O. Wachowiak M. (2008). Sniffing behavior of mice during performance in odor-guided tasks. Chem. Senses 33, 581–596. 10.1093/chemse/bjn029 18534995 PMC2533419

[B50] Xu A. Cui S. Wang J. (2015). Incoordination among subcellular compartments is associated to depression-like behavior induced by chronic mild stress. Inter. J. Neuropsychopharmacol. 19:pyv122. 10.1093/ijnp/pyv122 26506857 PMC4886664

[B51] Yan F. Gao Z. Chen P. Huang L. Wang D. Chen N.. (2016). Coordinated plasticity between barrel cortical glutamatergic and GABAergic neurons during associative memory. Neural Plast. 2016, 1–20. 10.1155/2016/5648390 28070425 PMC5192352

[B52] Ye B. Huang L. Gao Z. Chen P. Ni H. Guan S.. (2012). The functional upregulation of piriform cortex is associated with cross-modal plasticity in loss of whisker tactile inputs. PLoS ONE 7:e41986. 10.1371/journal.pone.0041986 22927919 PMC3424151

[B53] Yu J. Qian H. Chen N. Wang J. H. (2011). Quantal glutamate release is essential for reliable neuronal encodings in cerebral networks. PLoS ONE 6:e25219. 10.1371/journal.pone.0025219 21949885 PMC3176814

[B54] Yu J. Qian H. Wang J. H. (2012). Upregulation of transmitter release probability improves a conversion of synaptic analogue signals into neuronal digital spikes. Mol. Brain 5:26. 10.1186/1756-6606-5-26 22852823 PMC3497613

[B55] Zhang F. Liu B. Lei Z. Wang J. (2012). mGluR_1, 5_ activation improves network asynchrony and GABAergic synapse attenuation in the amygdala: implication for anxiety-like behavior in DBA/2 mice. Mol. Brain 5:20 10.1186/1756-6606-5-20 22681774 PMC3475049

[B56] Zhang G. Gao Z. Guan S. Zhu Y. Wang J. H. (2013). Upregulation of excitatory neurons and downregulation of inhibitory neurons in barrel cortex are associated with loss of whisker inputs. Mol. Brain 6:2. 10.1186/1756-6606-6-2 23286328 PMC3548736

[B57] Zhang M. Hung F. Zhu Y. Xie Z. Wang J. (2004). Calcium signal-dependent plasticity of neuronal excitability developed postnatally. J. Neurobiol. 61, 277–287. 10.1002/neu.20045 15382030

[B58] Zucker R. S. Regehr W. G. (2002). Short-term synaptic plasticity. Ann. Rev. Physiol. 25, 355–405. 10.1146/annurev.physiol.64.092501.114547 11826273

