# Supplementary material for: Coordinated Plasticity among Glutamatergic and GABAergic Neurons and Synapses in the Barrel Cortex Is Correlated to Learning Efficiency
Source: Front Cell Neurosci. 2017 Jul 26;11:221. doi: 10.3389/fncel.2017.00221 (PMC5526921; doi:10.3389/fncel.2017.00221)
Supplement: Supplementary file 1 [file Table1.DOC]

**Supporting Data to: Frontiers in Cellular Neuroscience**

**Coordinated plasticity among glutamatergic and GABAergic neurons and synapses in the barrel cortex is correlated to learning efficiency**

**Xin Zhao^1#^, Li Huang^1#^, Rui Guo^1^, Yulong Liu^1^, Shidi Zhao^1^, Sudong Guan^1^, Rongjing Ge^1^, Shan Cui^2^ and Jin-Hui Wang^1,2,3^***

***1) Department of Pathophysiology, Bengbu Medical College, Anhui 233000; 2) Institute of Biophysics and University of Chinese Academy of Sciences, Beijing China 100101; 3) Qingdao University, School of Pharmacy, 38 Dengzhou, Shandong China 266021***

Running title: Cell-specific mechanism for learning efficiency

Key words: learning, memory, glutamate, GABA, neuron, synapse and barrel cortex

**Corresponding author:**

Jin-Hui Wang, Ph.D. & MD

Brain and Cognitive Sciences

The Institute of Biophysics, Chinese Academy of Sciences

15 Datun Road, Beijing China 100101

[jhw@sun5.ibp.ac.cn](mailto:jhw@sun5.ibp.ac.cn); 86-10-64888472

Table 1A t and DF values of whisking frequencies in CR-formation mice with high efficiency and low efficiency as well as an unpaired control mice.

|  | Day 1 | | Day 2 | | Day 3 | | Day 4 | | Day 5 | | Day 6 | | Day 7 | | Day 8 | | Day 9 | | Day 10 | |
| --- | --- | --- | --- | --- | --- | --- | --- | --- | --- | --- | --- | --- | --- | --- | --- | --- | --- | --- | --- | --- |
|  | t | df | t | df | t | df | t | df | t | df | t | df | t | df | t | df | t | df | t | df |
| High vs. low | 0.04 | 240 | 0.87 | 240 | 2.11 | 240 | 3.47 | 240 | 3.51 | 240 | 4.62 | 240 | 4.03 | 240 | 3.01 | 240 | 1.20 | 240 | 0.67 | 240 |
| High vs. control | 0.04 | 240 | 0.92 | 240 | 3.08 | 240 | 5.11 | 240 | 6.41 | 240 | 7.42 | 240 | 7.94 | 240 | 8.11 | 240 | 8.60 | 240 | 9.17 | 240 |
| low vs. control | 0 | 240 | 0.052 | 240 | 0.97 | 240 | 1.634 | 240 | 2.9 | 240 | 2.80 | 240 | 3.91 | 240 | 5.10 | 240 | 7.39 | 240 | 8.5 | 240 |

 (n=9 mice for each group).

**Table 1B t and df** values of whisking angles in CR-formation mice with high efficiency and low efficiency as well as an unpaired control mice.

|  | Day 1 | | Day 2 | | Day 3 | | Day 4 | | Day 5 | | Day 6 | | Day 7 | | Day 8 | | Day 9 | | Day 10 | |
| --- | --- | --- | --- | --- | --- | --- | --- | --- | --- | --- | --- | --- | --- | --- | --- | --- | --- | --- | --- | --- |
|  | t | df | t | df | t | df | t | df | t | df | t | df | t | df | t | df | t | df | t | df |
| High vs. low | 0.01 | 144 | 2.27 | 144 | 4.85 | 144 | 6.05 | 144 | 6.26 | 144 | 6.37 | 144 | 5.97 | 144 | 5.11 | 144 | 3.40 | 144 | 2.73 | 144 |
| High vs. control | 0.28 | 144 | 4.29 | 144 | 8.30 | 144 | 10.84 | 144 | 13.26 | 144 | 15.35 | 144 | 16.51 | 144 | 16.7 | 144 | 17.71 | 144 | 17.74 | 144 |
| low vs. control | 0.29 | 144 | 2.04 | 144 | 3.44 | 144 | 4.78 | 144 | 7.00 | 144 | 8.98 | 144 | 10.54 | 144 | 11.59 | 144 | 14.31 | 144 | 15 | 144 |

(n=9 mice for each group).

**Table 2 t and DF values of the comparisons of sEPSC intervals and amplitudes at 67% cumulative probability on YFP-labeled glutamatergic neurons from high efficiency and low efficiency as well as an unpaired control mice**

|  | frequency | | amplitude | |
| --- | --- | --- | --- | --- |
|  | t | df | t | df |
| High vs. low | 6.33 | 28 | 2.865 | 26 |
| high vs. control | 12.471 | 28 | 4.344 | 26 |
| low vs. control | 6.45 | 28 | 2.253 | 26 |

(n=15 neurons from nine mice for each group)

|  | 1 | | 2 | | 3 | | 4 | | 5 | | 6 | | 7 | | 8 | | 9 | | 10 | | 11 | | 12 | | 13 | | 14 | |
| --- | --- | --- | --- | --- | --- | --- | --- | --- | --- | --- | --- | --- | --- | --- | --- | --- | --- | --- | --- | --- | --- | --- | --- | --- | --- | --- | --- | --- |
|  | t | df | t | df | t | df | t | df | t | df | t | df | t | df | t | df | t | df | t | df | t | df | t | df | t | df | t | df |
| High vs. low | 0.86 | 546 | 0.59 | 546 | 1.31 | 546 | 2.34 | 546 | 2.95 | 546 | 3.64 | 546 | 4.17 | 546 | 5.07 | 546 | 5.62 | 546 | 5.51 | 546 | 5.68 | 546 | 5.66 | 546 | 5.79 | 546 | 5.41 | 546 |
| High vs. control | 0.06 | 546 | 2.78 | 546 | 2.99 | 546 | 3.94 | 546 | 5.56 | 546 | 5.91 | 546 | 6.36 | 546 | 7.68 | 546 | 8.31 | 546 | 8.55 | 546 | 8.55 | 546 | 9.03 | 546 | 9.24 | 546 | 9.28 | 546 |
| low vs. control | 0.98 | 546 | 2.32 | 546 | 1.79 | 546 | 1.70 | 546 | 2.77 | 546 | 2.41 | 546 | 2.32 | 546 | 2.77 | 546 | 2.85 | 546 | 3.22 | 546 | 3.04 | 546 | 3.57 | 546 | 3.66 | 546 | 4.11 | 546 |

**Table 3 t and DF values of spikes per second versus normalized stimuli on YFP-labeled glutamatergic neurons in cortical slices with high efficiency and low efficiency as well as an unpaired control mice**

(n=15 neurons from nine mice for each group).

**Table 4 t and DF values of the comparisons of sIPSC intervals and amplitudes at 67% cumulative probability on YFP-labeled glutamatergic neurons from high efficiency and low efficiency as well as an unpaired control mice**

|  | frequency | | amplitude | |
| --- | --- | --- | --- | --- |
|  | t | df | t | df |
| High vs. low | 8.748 | 23 | 5.267 | 25 |
| high vs. control | 16.075 | 24 | 6.46 | 25 |
| low vs. control | 7.968 | 23 | 3.05 | 25 |

(n=15 neurons from nine mice for each group)

**Table 5 t and DF values of the comparisons of sEPSC intervals and amplitudes at 67% cumulative probability on GFP-labeled GABAergic neurons from high efficiency and low efficiency as well as an unpaired control mice**

|  | frequency | | amplitude | |
| --- | --- | --- | --- | --- |
|  | t | df | t | df |
| High vs. low | 8.328 | 28 | 2.865 | 28 |
| high vs. control | 19.074 | 28 | 4.344 | 25 |
| low vs. control | 7.348 | 28 | 2.253 | 25 |

(n=15 neurons from nine mice for each group)

**Table 6 t and DF values of spikes per second versus normalized stimuli on GFP-labeled GABAergic neurons in cortical slices with high efficiency and low efficiency as well as an unpaired control mice**

|  | 1 | | 2 | | 3 | | 4 | | 5 | | 6 | | 7 | | 8 | | 9 | | 10 | | 11 | | 12 | | 13 | | 14 | |
| --- | --- | --- | --- | --- | --- | --- | --- | --- | --- | --- | --- | --- | --- | --- | --- | --- | --- | --- | --- | --- | --- | --- | --- | --- | --- | --- | --- | --- |
|  | t | df | t | df | t | df | t | df | t | df | t | df | t | df | t | df | t | df | t | df | t | df | t | df | t | df | t | df |
| High vs. low | 0.47 | 336 | 0.42 | 336 | 1.33 | 336 | 1.91 | 336 | 2.19 | 336 | 2.63 | 336 | 3.00 | 336 | 3.26 | 336 | 3.44 | 336 | 3.96 | 336 | 3.76 | 336 | 4.02 | 336 | 4.06 | 336 | 4.30 | 336 |
| High vs. control | 0.84 | 336 | 2.84 | 336 | 5.60 | 336 | 6.68 | 336 | 7.40 | 336 | 8.34 | 336 | 9.13 | 336 | 9.48 | 336 | 9.76 | 336 | 10.48 | 336 | 10.37 | 336 | 10.7 | 336 | 10.65 | 336 | 10.78 | 336 |
| low vs. control | 1.23 | 336 | 2.34 | 336 | 4.18 | 336 | 4.70 | 336 | 5.16 | 336 | 5.65 | 336 | 6.09 | 336 | 6.18 | 336 | 6.29 | 336 | 6.53 | 336 | 6.60 | 336 | 6.68 | 336 | 6.60 | 336 | 6.51 | 336 |

(n=15 neurons from nine mice for each group)
